# NanoFAST: structure-based design of a small fluorogen-activating protein with only 98 amino acids†

**DOI:** 10.1039/d1sc01454d

**Published:** 2021-04-08

**Authors:** Konstantin S. Mineev, Sergey A. Goncharuk, Marina V. Goncharuk, Natalia V. Povarova, Anatolii I. Sokolov, Nadezhda S. Baleeva, Alexander Yu. Smirnov, Ivan N. Myasnyanko, Dmitry A. Ruchkin, Sergey Bukhdruker, Alina Remeeva, Alexey Mishin, Valentin Borshchevskiy, Valentin Gordeliy, Alexander S. Arseniev, Dmitriy A. Gorbachev, Alexey S. Gavrikov, Alexander S. Mishin, Mikhail S. Baranov

**Affiliations:** Institute of Bioorganic Chemistry, Russian Academy of Sciences Miklukho-Maklaya 16/10 117997 Moscow Russia baranovmikes@gmail.com; Moscow Institute of Physics and Technology Dolgoprudny 141701 Russia; Institute of Biological Information Processing (IBI-7: Structural Biochemistry), Forschungszentrum Jülich GmbH Jülich 52425 Germany; JuStruct: Jülich Center for Structural Biology, Forschungszentrum Jülich GmbH Jülich 52425 Germany; ESRF – The European Synchrotron Grenoble 38000 France; Institut de Biologie Structurale J.-P. Ebel, Université Grenoble Alpes, CEA, CNRS Grenoble France; Pirogov Russian National Research Medical University Ostrovitianov 1 Moscow 117997 Russia

## Abstract

One of the essential characteristics of any tag used in bioscience and medical applications is its size. The larger the label, the more it may affect the studied object, and the more it may distort its behavior. In this paper, using NMR spectroscopy and X-ray crystallography, we have studied the structure of fluorogen-activating protein FAST both in the apo form and in complex with the fluorogen. We showed that significant change in the protein occurs upon interaction with the ligand. While the protein is completely ordered in the complex, its apo form is characterized by higher mobility and disordering of its N-terminus. We used structural information to design the shortened FAST (which we named nanoFAST) by truncating 26 N-terminal residues. Thus, we created the shortest genetically encoded tag among all known fluorescent and fluorogen-activating proteins, which is composed of only 98 amino acids.

## Abstract 2

We solved the structure of a fluorogen-activating protein FAST and synthesized the library of potential fluorogens. Using these data, we designed the shortest genetically encoded fluorescent tag among all known.

## Graphical abstract

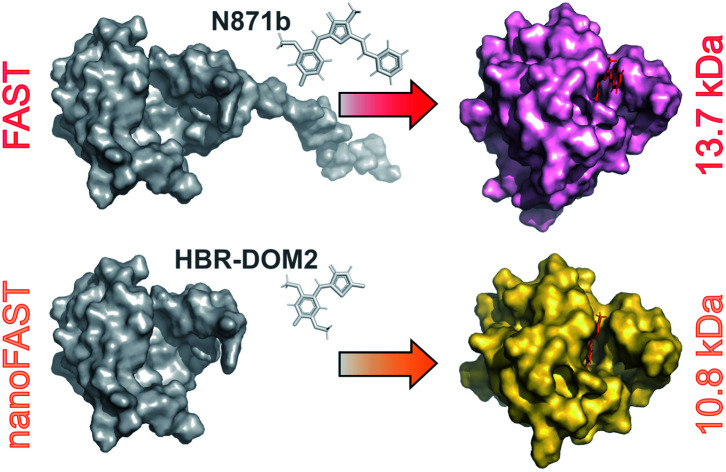

## Introduction

Various fluorescent tags have long and widely been used in modern biomolecular research.^1^ Over the past decades, many such tags have been developed, mostly fluorescent proteins (FP),^2^ which are genetically encoded tags formed solely from the internal components of a biological object. Alternatively, completely external chemical tags can also be used.^3^ However, the greatest interest in recent years has been acquired by the combined tags, with one of the components being internally genetically encoded, while the second, small-molecule component is supplied from the outside. The best known labels of this kind are two-component Halo-,^4^ and SNAP-tags^5^ or three-component system based on mutant forms of lipoic acid ligase.^6^

Nevertheless, all these tags have a number of drawbacks. Fluorescent proteins are quite large and require a considerable time and the presence of oxygen for maturation,^7^ while the use of chemical fluorescent dyes in any role often leads to off-target labeling.^8^

In this regard, the approaches that employ the so-called fluorogens – substances with very weak fluorescence in the free state, which become bright only when they reversibly bind to the tag's secondary component look more promising. The internal component of such labels can be nucleic acid^9^ or fluorogen-activating protein (FAP).^10^ Such tags do not require oxygen and can be used under anaerobic conditions. Their maturation time is small and corresponds to the time of protein folding, while the fluorescent signal can be induced or removed on demand by simple addition or washing out of fluorogen.^11^ The size of the tag is also an important characteristic. The larger it is, the more it affects the natural dynamics of the tagged protein.^12^ Apart from their other advantages, FAPs are almost two times smaller than FP. Nevertheless, the size of such proteins is still about 120–150 amino acids while the shorter FAPs either are too dim to be used for imaging or require huge fluorogens with poor membrane permeability.^13^ Properties of several examples of such tags are presented in Table 1.

**Table 1:** Optical properties of various FAPs and FPs

FP of FAP	Size, kDa	Absorbance maxima position, nm	Emission maxima position, nm	*ε*, M^−1^ cm^−1^	Fluorescence quantum yield (FQY), %	Brightness (=*ε*·FQY)	*K*_d_, μM
GFP^2^	26.9	488	507	55 900	60	33 500	—
mNeonGreen^24^	26.6	506	517	116 000	80	92 800	—
mOrange2 (ref. 2)	26.8	549	565	58 000	60	34 800	—
mFAP1:DFHBI^13*a*^	12.2	485	506	49 300	2.1	1000	0.56
L5-MG:MG-2p^13*b*^	11.5	640	668	103 000	4.8	5000	0.32
DiB1 (ref. 11*a*)	19.7	513	542	45 800	32	14 650	0.1
FAST:**HMBR**^14,19^	13.6	483	540	45 000	33	14 850	0.13
FAST:**HBR-DOM**^14,19^	13.6	520	600	39 000	31	12 000	0.97
FAST:**N871b**^19^	13.6	562	606	23 000	25	5750	0.25
FAST:**HBR-DOM2**	13.6	510	566	30 500	54 ± 3	16 500	0.021 ± 0.002
nanoFAST:**HBR-DOM2**	10.8	502	563	25 500	56 ± 3	14 300	0.85 ± 0.05

One of the most promising protein among various FAPs is the so-called FAST protein (“Fluorescence-Activating and absorption-Shifting Tag”),^14^ an engineered variant of the photoreceptor from *Halorhodospira halophila* – Photoactive Yellow Protein (PYP).^15^ This photoactive protein covalently binds a hydroxycinnamic acid. However, the replacement of several amino acids (including the key cysteine responsible for covalent binding) allowed using it as an FAP with a group of fluorogens. Over the past five years, a series of multi-colored variants^16^ and split constructs^17^ have been created based on this FAP.

Nevertheless, the structure of this protein and its complexes was unknown. It was only evident that the interaction with fluorogens resembled the binding of the native ligand of the PYP, since in the FAST : fluorogen complex, the phenolic fragment of the fluorogen was deprotonated, probably due to interaction with amino acids E46 and Y42.^18^

In this work, we have used NMR spectroscopy to study the structure of both the FAST apo form and its complex with the previously proposed fluorogen **N871b** (Fig. 4).^19^ We have shown that significant change in the protein occurs upon interaction with the ligand. Using the data, we found that the N-terminally truncated variant of FAST protein (which we named nanoFAST) can also be used as an FAP.

## Results and discussion

**Fig. 1:**
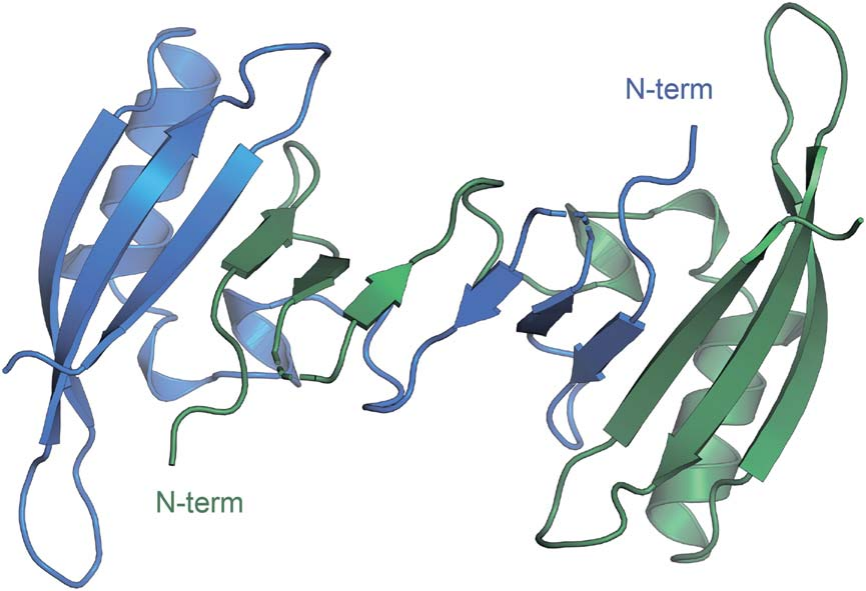
Crystal structure of FAST in the domain-swapped dimer form.

Through the whole of our study, we crystallized the FAST protein several times, either in the presence of various ligands or without them. Surprisingly, we found that the protein adopts a form of the ligand-free domain-swapped dimer in crystals under a wide variety of conditions. In this dimer, the first three strands of the core β sheet (A30-L33, I39-N43, and T50-R52) are exchanged with the corresponding elements from the symmetrical molecule, forming together one large twelve-stranded β sheet (Fig. 1, ESI Part 3†).

To further investigate the FAST ligand binding, we produced the ^13^C/^15^N isotope-labeled FAST and solved the spatial structure of the protein in complex with a promising ligand **N871b** previously proposed by us^19^ and in the apo state using NMR spectroscopy (ESI Part 4†). Contrary to X-ray data and in good correlation with reported previously,^14^ both forms of protein are present in solution exclusively in the monomeric form (the determined hydrodynamic radius was 2.1 ± 0.1 nm, which corresponds to the 15 kDa). Initial characterization of FAST-apo revealed the poor quality of NMR spectra due to the enhanced slow conformational transitions (Fig. S4.1–S4.3†). In contrast, ligand binding stabilized the protein substantially, providing a perfect NMR spectrum. Thus, we first investigated the structure of FAST in complex with **N871b**.

**Fig. 2:**
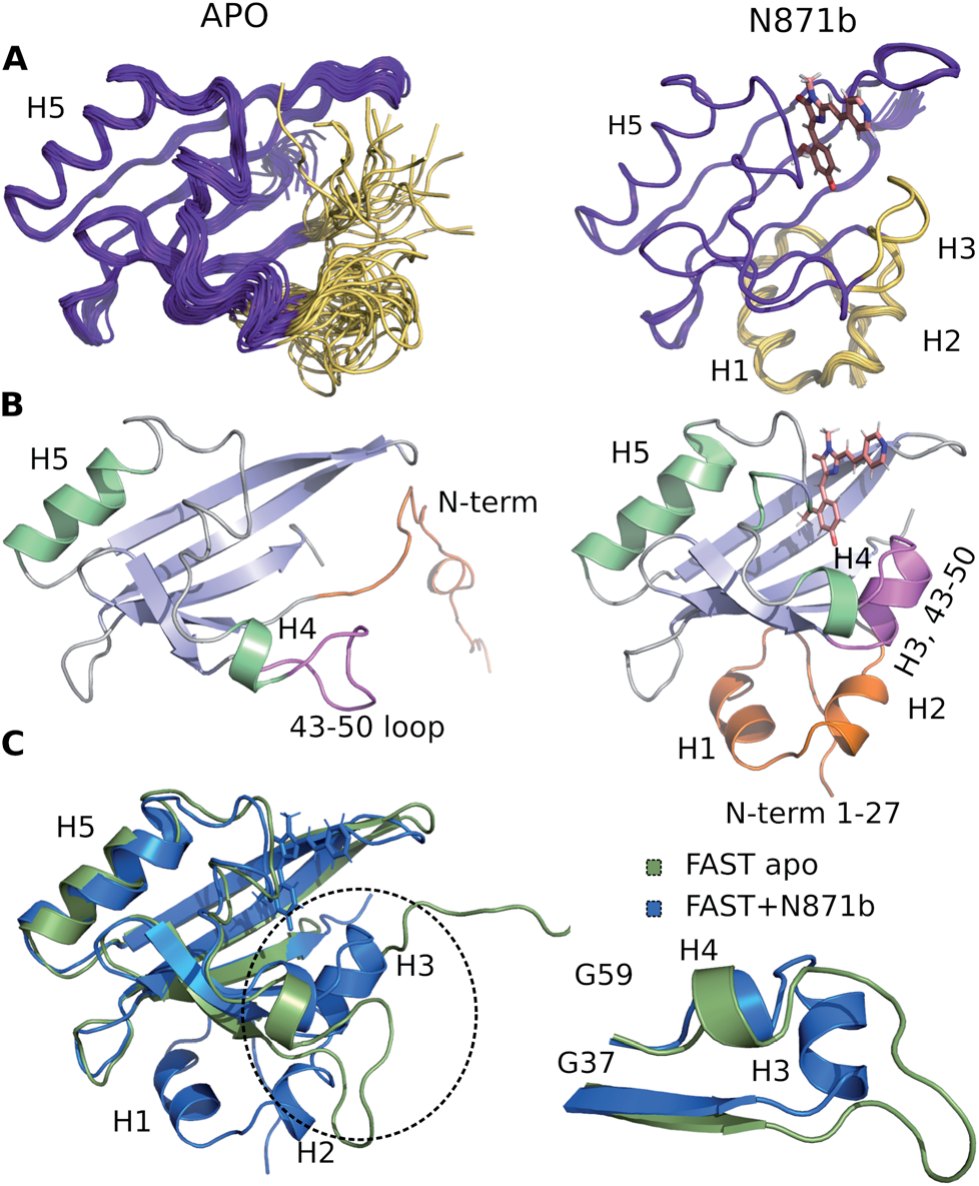
3D structure of FAST-apo (A and B left, superposition on C) and FAST:**N871b** complex (A and B right, superposition on C). (A) 20 best NMR structures, superimposed over the backbone atoms of the secondary structure elements. N-terminus and region 43–50 that change the structure upon ligand binding are shown in ivory. (B) Representative structures. α-Helices are shown in green and β-strands are shown in blue. N-terminus and region 43–50 are highlighted by orange and magenta, respectively. (C) Structures of FAST-apo (green) and FAST:**N871b** (blue), superimposed over the backbone atoms of 5 β-strands and helix H5. Unstructured C-terminal His6 tag is not shown.

The high quality of NMR data allowed obtaining 97% of possible chemical shift assignment and determining the structure in a semi-automated manner, with the intermolecular distances being observed directly *via* the isotope-filtered experiments (Fig. S4.4†).^20^ In complex with **N871b**, FAST reveals the typical architecture of a PAS domain,^21^ similar to the fold of its parent PYP protein (Fig. 2).^22^ According to the PDBeFOLD server, the backbone atoms of the complex may be superimposed with the coordinates of PYP (PDBID 1F98 (ref. 22)) with the RMSD of 1.16 Å.

**Fig. 3:**
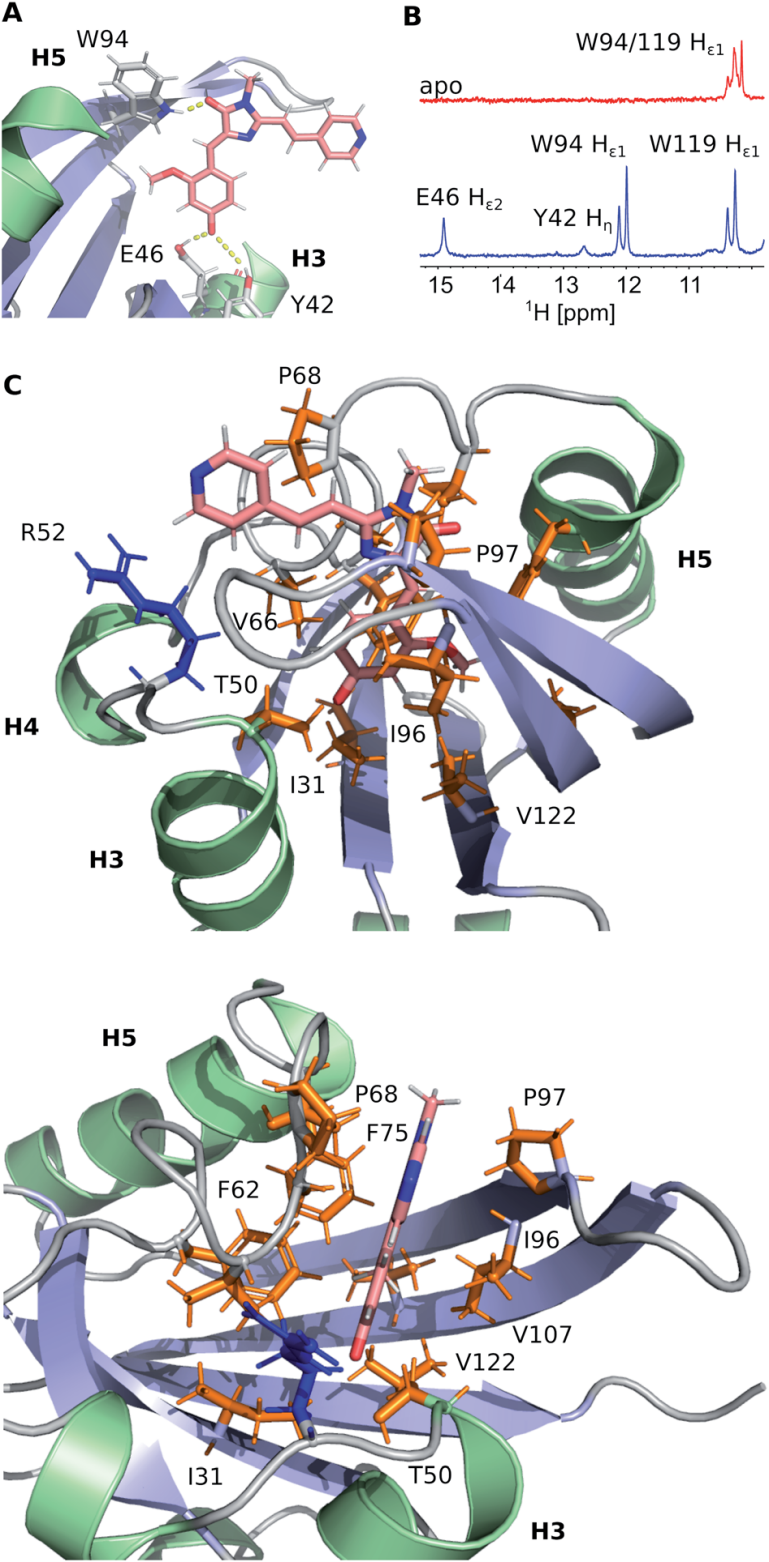
Details of the **N871b** binding by FAST. (A) Hydrogen bonds involved in **N871b** binding. (B) A low-field region of ^1^H NMR spectra of FAST in the apo state (red) and in complex with **N871b** (blue). Both spectra were recorded at pH 7.0, 25 °C. The assignment of protons is indicated. (C) Non-bonding interactions, detected within the FAST:**N871b** complex. Sidechains of hydrophobic residues are painted in orange, R52 positively charged sidechain is shown in blue.

FAST chain forms a 5-stranded β-sheet and 5 α-helices, and the ligand is placed inside a hydrophobic cavity, stabilized by three hydrogen bonds (Fig. 3). Like 4-hydroxycinnamic acid residue in PYP, the oxygen of **N871b** phenol ring forms an H-bond with the protonated sidechain of E46 (H_ε2_) and a more distant polar contact with phenol group of Y42 (H_η_), which is supported by the presence of two broad low-field peaks in ^1^H NMR spectra of these labile protons (Fig. 3). In addition, the carbonyl group of a 5-member ring is engaged in an H-bond with the ε1-imino-group of W94. Besides, the ligand binding is favored by the interactions with hydrophobic sidechains of I31, T50, V66, A67, P68, T70, I96, P97, V107 and V122 and π-stacking with the rings of F62 and F75. The ligand molecule is larger than the cavity, and it exits the protein near the C-terminus of helix 3, with the direct contact of the solvent-exposed pyridine group with the positively charged R52 sidechain.

To investigate the structure of ligand-free FAST, we had to heat the protein to 45 °C and use a lower-field NMR magnet. Together, these two actions allowed reducing the effect of slow conformational transitions and solving the spatial structure (Table S5†). The initial analysis revealed a drastic difference between the apo and bound states of FAST (Fig. 2 and S4.5†). The whole N-terminal part, which includes helices H1 and H2 appeared completely disordered in the apo state. Region of helix H3, containing three residues, engaged in the ligand binding in PYP and FAST:**N871b** complex, became unstructured. However, the coordinates of the remaining elements of secondary structure – a 5-stranded β-sheet and helix H5 remain unchanged with respect to the FAST:**N871b** complex and could be superimposed with the RMSD as small as 0.9 Å. Thus, the ligand binding to FAST induces the formation of helix H3 and stabilizes the N-terminal residues.

Since the ligand binding should begin with the initial apo form before the protein rearrangement, we hypothesized that the binding can occur in the absence of N-terminus and the shortened FAST can retain fluorogen-activating properties.

**Fig. 4:**
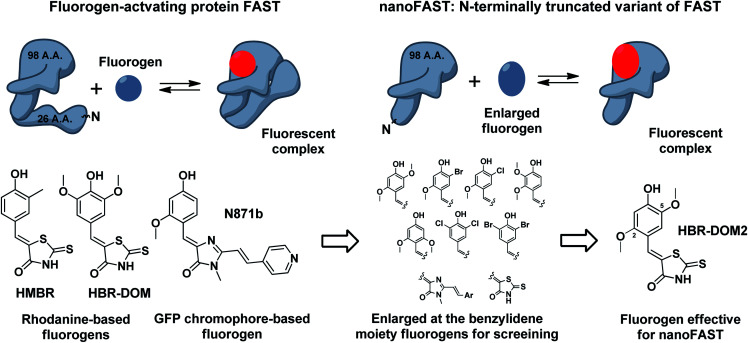
Principal scheme of FAST protein action revealed by NMR analysis, proposed nanoFAST protein and their fluorogens.

Thus, next, we created an N-terminally shortened variant of FAST protein (truncated up to the 26th residue – nanoFAST). We found that it is inactive against such known fluorogens as **HMBR**, **HBR-DOM**, **N871b**, and others (ESI, Part 5†). Since the protein pocket is slightly enlarged in the apo form, the pocket of nanoFAST also should be bigger than in the original FAST. Thus, at the next stage, we created a library of compounds with enlarged benzylidene moiety (Fig. 4, ESI Part 9†).

The screening of this library (ESI Part 5†) showed that the introduction of additional bulky substituents allows recovering the ligand binding with the protein. It turned out to be most effective in the case of 2.5 disubstituted substances, and especially 2.5 dimethoxy rhodanine – compound **HBR-DOM2**. This substance's fluorescence intensity increased by more than a hundred times upon interaction with the nanoFAST protein. The fluorescence quantum yield of the complex reached 55%, while the binding constant turned out to be close to 1 μM, which is similar to the characteristic of previously obtained pairs with FAST (Table 1, ESI Parts 6 and 7†).

**Fig. 5:**
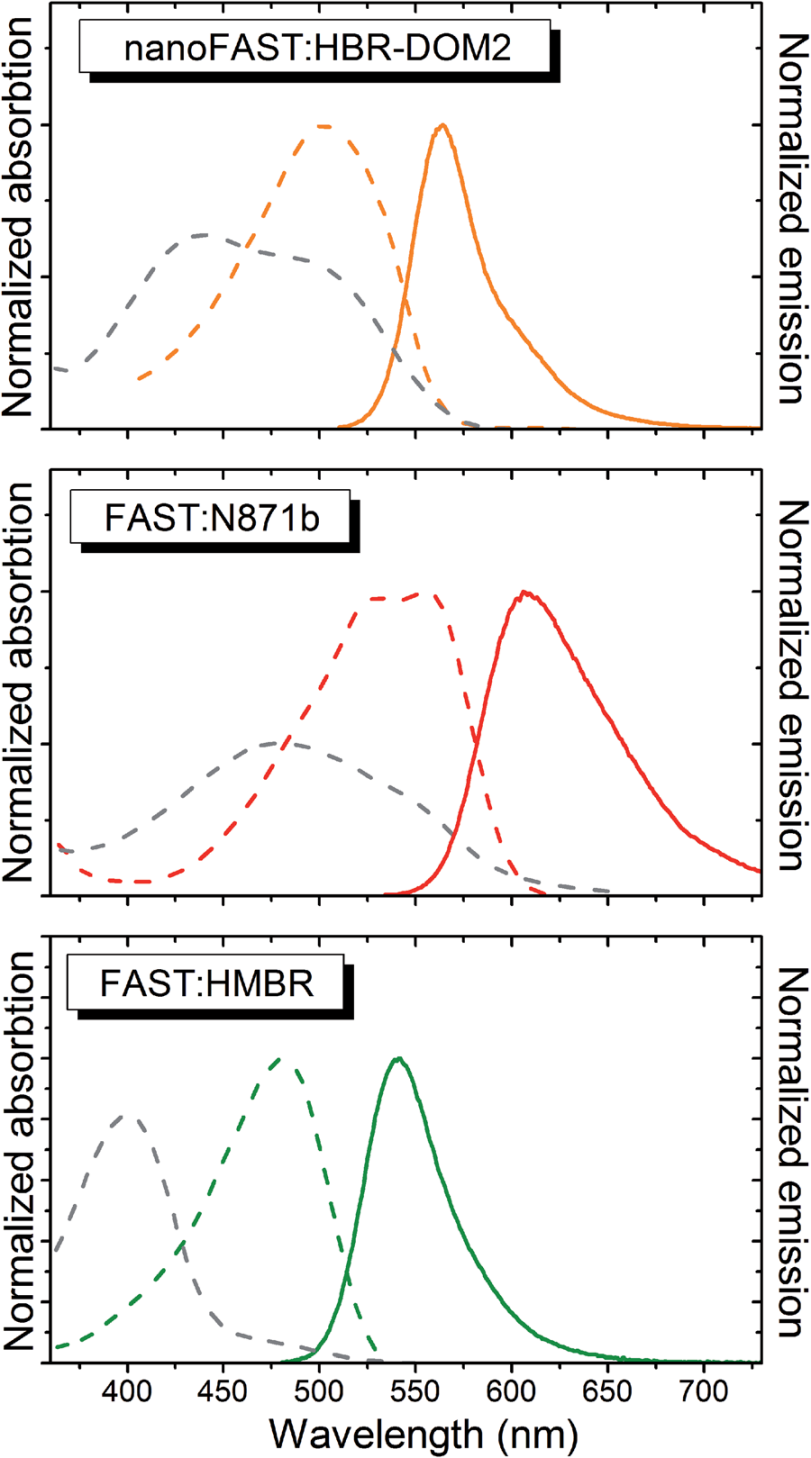
The absorption (dashed lines) and emission (solid line) spectra of free fluorogens (grey) and protein : fluorogen complexes (colored). The ratio of the absorption intensity of the complex and free fluorogen corresponds to the real change in the extinction coefficient and shape of the spectra in PBS. The curves are from a single measurement.

The absorption and emission spectra of the obtained complex lay between the spectra of complexes of FAST protein with fluorogens **N871b** and **HMBR** (Fig. 5 and Table 1). The fluorogen **HBR-DOM2** also efficiently binds to the original FAST protein with a more than an order of magnitude lower constant and a similar orange fluorescence color. The changes in the spectra occurring upon the **HBR-DOM2** binding to FAST and nanoFAST also reveal the deprotonation of its phenolic moiety. In a protein-free form, this fluorogen is already partially deprotonated at neutral pH; however, the low quantum yield of fluorescence of a free form allows avoiding an off-target signal.

**Fig. 6:**
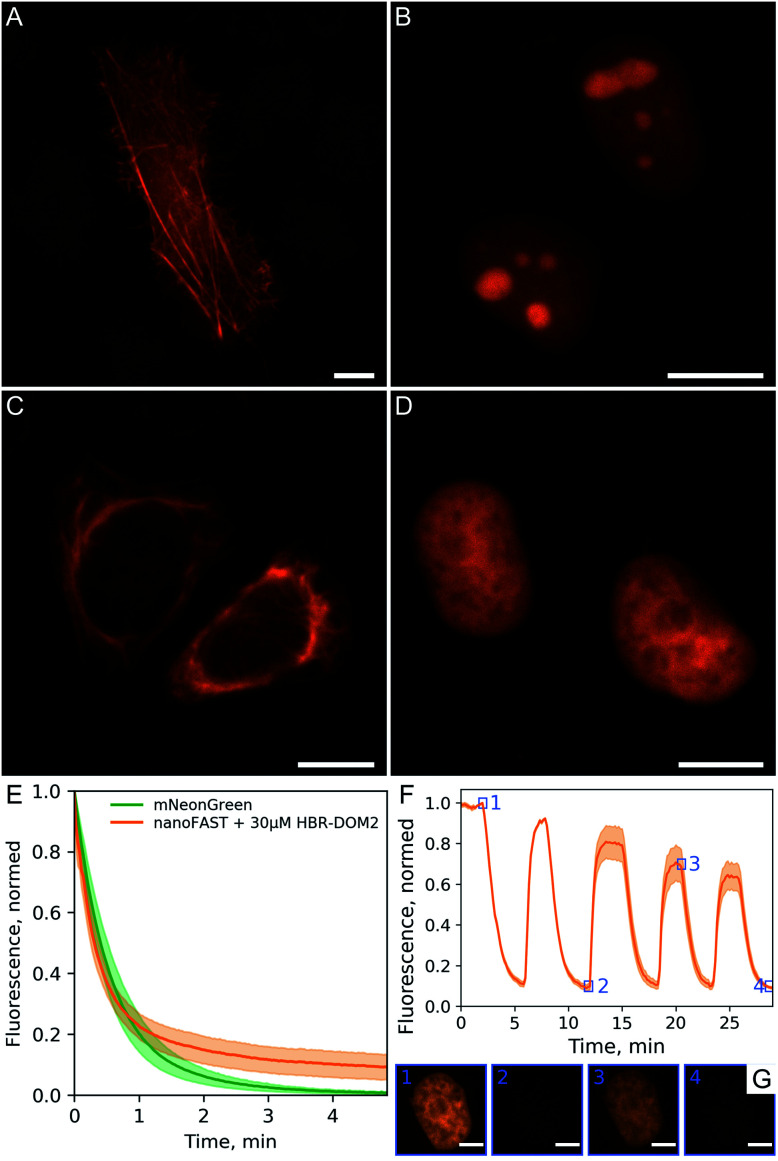
Performance of fluorogenic complex of nanoFAST with **HBR-DOM2** in fluorescence microscopy. Fluorescence imaging of live U2OS cells transiently transfected with (A) lifeact-nanoFAST and live HeLa cells transiently transfected (B) 3 × NLS-nanoFAST, (C) vimentin-nanoFAST and (D) H2B-nanoFAST constructs in the presence of 5 μM **HBR-DOM2**; scale bars are 10 μm. (E) Photobleaching of nanoFAST in the presence of 30 μM **HBR-DOM2** in comparison with mNeonGreen under confocal microscopy conditions. Solid lines represent mean value ± SD (shaded, *n* = 10 nuclei for each construct) (F) sequential staining and washout of live HeLa cells transiently transfected with H2B-nanoFAST construct; 5 μM concentration of **HBR-DOM2** was used for staining; solid line – mean value ± SD (shaded, *n* = 4 nuclei) individual frames of single labeled nuclei from timings indicated by numbered blue squares presented on panel (G); scale bars are 5 μm.

We demonstrated the efficiency and utility of the proposed pair on a series of cells transiently transfected with various nanoFAST fusions (Fig. 6A–D, ESI Part 8†).

The fluorescent signal of the resulting complex turned out to be sufficiently photostable. We compared it with the mNeonGreen fluorescent protein,^24^ which has a similar absorption spectrum and showed that their photobleaching curves behave in a similar way (Fig. 6E). We have also confirmed that the binding of our fluorogen to the nanoFAST protein is non-covalent and, if necessary, it can be easily washed out from cell media (Fig. 6F, ESI Video†).

## Conclusions

In this work we report the first spatial structures of fluorogen-activating protein FAST in both the ligand-bound and ligand-free states. Initial design of FAST,^14^ as well as several most recent works reporting the enhanced FAST mutants^16,25^ were performed utilizing the blind directed evolution/random mutagenesis approaches. While the similarity between the PYP and FAST conformations was implied, the determinants of ligand binding were still unknown. Now, the resolved structure of the FAST:**N871b** complex paves the way to the structure-guided rational engineering of the FAST variants with the improved stability and optical properties, and explains the effects of previously suggested mutations. The intermolecular hydrogen bond, formed by the indole group of W94 accounts for the essential role of ^94^W[x]IPT fragment, elucidated in the course of the initial directed evolution experiment. The detailed analysis of protein : ligand packing reveals that some other known FAST mutations, such as V107I^25*b*^ and F62L,^16^ correspond to the residues in the direct contact with the 4-hydroxy-3-methoxyphenyl moiety of the ligand, and may improve the packing and stability of the protein : ligand complex. M95T^25*a*^ mutation is proximal to the W94, which forms the key hydrogen bond and S99K^25*a*^ substitution may result in the favorable electrostatic contact with the rhodanine moiety of the ligand. Previously the high-throughput directed evolution approaches were applied successfully to improve the FAPs.^16,25^ Nonetheless, random mutagenesis does not allow sampling all possible protein variants, moreover some beneficial mutations may be lost due to the decreased protein synthesis level in cells or other problems. Therefore, the possibility of the structure-guided rational design of the protein is highly important.

The structure of FAST in the apo-form is equally valuable. Since there was no data present on the conformation of PYP in the absence of its covalently bound fluorophore, this ligand-free state of FAST was a complete mystery. As we show here, several important elements of FAST structure are lost in the apo state. Namely, two N-terminal helices and a small third helix become unstructured. Surprisingly, similar changes were found to take place in the light-induced state of PYP previously.^23^ The most important observation in the context of this work is that 27 N-terminal residues of FAST do not contact directly with the ligand and are unstructured in the apo state of the protein. This led us to the idea that the N-terminus of FAST protein can be removed without the loss of fluorogen-activating properties.

Initial results of such truncation were rather upsetting, since all the previously reported FAST ligands stopped working with the shortened FAST variant, which we named nanoFAST. Thus, next we synthesized the vast library of novel possible ligands. As a result, we revealed one efficient fluorogene – compound **HBR-DOM2**. We showed that nanoFAST:**HBR-DOM2** pair can be used for the bright and reversible fluorescent labeling of various protein fusions in the living cells. The presented fluorogen-activating protein nanoFAST is only 98 amino acid long and appears the smallest out of all known protein-based fluorescent tags.

Altogether, we provide here an example of successful bidirectional structure-based rational design of a FAP : fluorogen pair.

## Author contributions

K. S. M. resolved the NMR structures of FAST and wrote the manuscript. S. A. G., M. V. G. expression and purification of ^13^C/^15^N isotope-labeled FAST. A. S. A. helped with research design and advice. A. I. S., N. S. B., A. Yu. S., I. N. M. synthesis and characterization of chromophore library. S. B., A. R., A. M., V. B., V. G. crystallization, collection and analysis of X-ray data, writing of the relevant parts of the manuscript. D. A. R., N. V. P. expression and purification of non-labeled FAST and nanoFAST. D. A. G., A. S. G. living cell transfection and fluorescent microscopy. A. S. M. helped with research design and advice, writing the manuscript. M. S. B. supervised the project, provided advice and wrote the manuscript.

## Conflicts of interest

There are no conflicts to declare.

## Supplementary Material

SC-012-D1SC01454D-s001

SC-012-D1SC01454D-s002
